# ELF5 and DOK7 regulation in anti-estrogen treated cells and tumors

**DOI:** 10.1186/s12935-016-0282-9

**Published:** 2016-02-16

**Authors:** Lily M. Fitzgerald, Eva P. Browne, Kevin D. Christie, Elizabeth C. Punska, Leo O. Simmons, Kristin E. Williams, Brian T. Pentecost, Rahul M. Jawale, Christopher N. Otis, Kathleen F. Arcaro

**Affiliations:** Department of Environmental Science, University of Massachusetts Amherst, Amherst, MA USA; Department of Veterinary and Animal Science, University of Massachusetts Amherst, Amherst, MA USA; Department of Biochemistry and Molecular Biology, University of Massachusetts Amherst, Amherst, MA USA; Department of Molecular and Cellular Biology, University of Massachusetts Amherst, Amherst, MA USA; Wadsworth Center, New York State Department of Health, Albany, NY USA; Pathology Department, Baystate Medical Center, Springfield, MA USA

**Keywords:** Breast cancer, DOK7, ELF5, Estrogen receptor, Progesterone receptor, Tamoxifen resistance

## Abstract

**Background:**

Most women with primary breast cancers that express estrogen receptor alpha (ER or ESR1) are treated with endocrine therapies including the anti-estrogen tamoxifen, but resistance to these anti-endocrine therapies often develops. This study characterizes the expression of hormone receptors, and the mRNA and DNA methylation levels of docking protein 7 (DOK7), and E74-like factor 5 (ELF5), in 21 novel tamoxifen-resistant cell lines and extends the findings to primary and recurrent human breast tumors.

**Methods:**

Twenty-one tamoxifen-selected cell lines were developed through cloning by limiting dilution of an MCF-7 cell culture treated with 1 μM tamoxifen for 6 months. The parent (MCF-7) and tamoxifen-selected cell lines were characterized for protein expression of ER, progesterone receptor (PR) and human epidermal growth factor receptor 2 (HER2) using immunohistochemistry (IHC). The mRNA levels of ER, DOK7, and ELF5 were assessed using quantitative RT-PCR. Promoter methylation levels of DOK7 and ELF5 were determined by pyrosequencing of bisulfite-modified DNA. The relationship between hormone receptor status and promoter methylation of DOK7 and ELF5 was further examined using available methylation array data (Illumina HM450) from a set of paired primary and second breast tumors from 24 women.

**Results:**

All 21 of the novel tamoxifen-selected cell lines are ER-positive, and HER2-negative, and 18 of the cell lines are PR-negative while the MCF-7 cells were scored as ER-positive, modestly PR-positive and HER2 negative. Expression of DOK7 and ELF5 is significantly up-regulated in half of the tamoxifen-selected cell lines as compared to the parental MCF-7. In contrast, the previously established ER-negative TMX2-28 cell line has decreased expression of both DOK7 and ELF5 and increased DNA methylation in the transcriptional start site region of these genes. ELF5 methylation was lower in second versus primary tumors in women who received anti-estrogen treatment, in PR-negative versus PR-positive tumors, and in the subset of PR-positive first tumors from the group of women who had second PR-negative tumors as compared to those who had second PR-positive tumors.

**Conclusions:**

The distinct ELF5 methylation of PR-positive primary tumors from women who had a PR-negative recurrence indicates the possibility of stratification of women for tailored treatment in the early stages of disease.

## Background

Approximately 80 % of primary breast cancers express estrogen receptor α (ER), a nuclear transcription factor that is the product of the *ESR1* gene [1–3]. Aromatase inhibitors and anti-estrogens such as tamoxifen are used to treat these estrogen receptor positive (ER+) cancers, and act to reduce the growth-promoting effects of estrogen. However, 33 % of women treated with tamoxifen see a recurrence in their breast cancer within 5 years, and resistance to all endocrine therapies is common [4]. Many potential molecular changes could allow breast cancer cells to continue growth in the absence of estrogen, and the acquired drug resistance is incompletely understood. Mutations to the *ESR1* gene [1, 5], chromatin restructuring [6], and disruption of many cellular pathways [7, 8] have been described in endocrine resistant breast cancers, as reviewed [9–11]. We have previously shown that tamoxifen treatment alters DNA methylation of various genes involved in cell growth in breast cancer cell lines [12].

Despite their insensitivity to tamoxifen, most resistant breast cancers still express ER [9]. Resistant cell lines developed in previous experiments were primarily ER+, with exceptions such as the triple negative, TMX 2-28 [13, 14]. The characterization of our novel cell lines began with measuring the intracellular protein levels of three receptors: ER, progesterone receptor (PR), and human epidermal growth factor 2 (HER2). The intracellular protein levels of ER, PR, and HER2 in breast cancers often advise the treatment choices for the patient. Cancers with decreased levels of PR are more likely to become resistant to endocrine therapy [15], and HER2 overexpression is known to promote tamoxifen resistance via cross-talk with ER [16].

Due to the frequency of acquired endocrine resistance and the lack of treatment for this type of breast cancer, many researchers have studied biological mechanisms that predict acquired endocrine resistance. One recently recognized marker of acquired endocrine resistance is E74-liked factor 5 (ELF5) [8]. ELF5 is a transcription factor involved in keratinocyte and mammary gland differentiation, especially alveolar differentiation, milk secretion and ductal morphogenesis [17]. ELF5 is frequently down-regulated in breast cancer [18]. A recent study identified ELF5 as playing a potential role in the development of tamoxifen resistance that is positively correlated to ER expression [8]. Progesterone has been shown to induce ELF5 levels, and the action of progesterone on ELF5 expression in alveolar development is thought to be via a paracrine mechanism [19]. For the above reasons, ELF5 expression in each of our tamoxifen-selected novel cell lines was compared to that in MCF-7, as well as that in the previously established tamoxifen-resistant cell lines: TMX2-4, TMX2-11 and TMX2-28.

Additionally, expression and methylation of docking protein 7 (DOK7) were measured due to the protein’s potential role as a tumor suppressor. A recent study of identical twins with differential breast cancer statuses identified DOK7 gene methylation as an indicator of breast cancer risk [20]. Increased expression of DOK7 has been correlated with longer patient survival time, and decreased expression of the gene has been seen in patients with recurrent cancers [21]. To our knowledge, a role of DOK7 in tamoxifen resistance has not been previously studied.

In this study, we sought to characterize tamoxifen-selected MCF-7 derivative cell lines based on changes in the expression and methylation of DOK7, ELF5, and ERα, and the protein levels of ERα, PR, and HER2. To extend these findings clinically, we examined the relationship between hormone receptor status, anti-estrogen treatment and promoter methylation of DOK7 and ELF5 in a set of matched primary and recurrent breast tumors from 24 women.

## Results and discussion

### Novel tamoxifen-selected cell lines phenotypically resemble the parental line

**Fig. 1:**
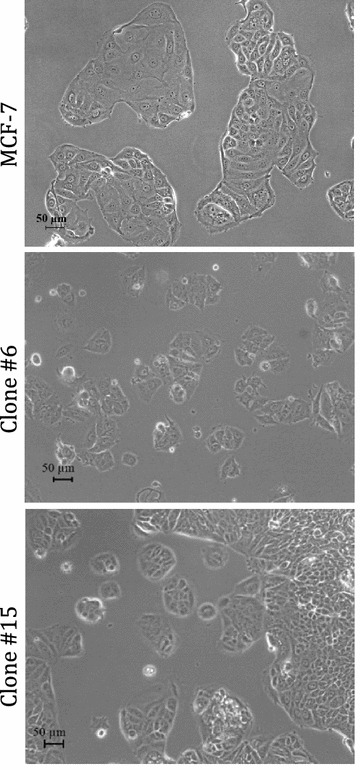
Phenotypic characterization of tamoxifen-selected cell lines. Representative phase-contrast photomicrographs of the parental MCF-7 (*top panel*) and two clonal cell lines: Tamoxifen-selected clone #6 (*middle panel*) and tamoxifen-selected clone #15 (*bottom panel*). Images were taken at ×25 original magnification and *scale bar* = 50 μM. All clonal cell lines were phenotypically similar to the parental MCF-7 cell line and grew at similar rates

During the initial expansion of the tamoxifen-selected cell lines, a few of the cell lines appeared to grow either in unusual clumps or in suspension. However, after several passages in T-75 flasks all of the tamoxifen-selected lines displayed a phenotype similar to that of MCF-7 (Fig. 1).

### Novel tamoxifen-selected cell lines are ER-positive and PR- and HER2-negative

**Fig. 2:**
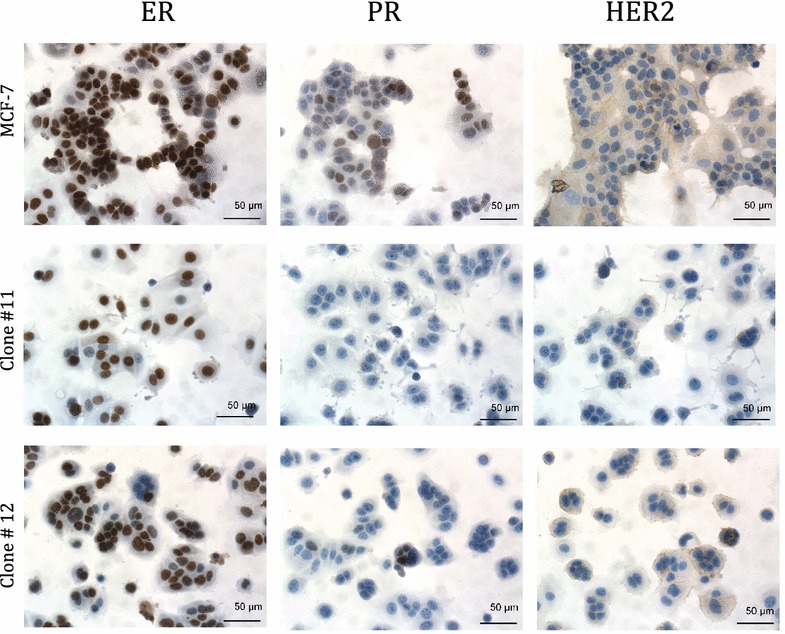
Hormone receptor status of cell lines. Immunohistochemistry results for estrogen receptor (ER), progesterone receptor (PR) and human epidermal growth factor receptor 2 (HER2) in MCF-7, and a representative tamoxifen-selected clonal cell line (clone 3) are shown. Images were taken at ×200 original magnification. PR staining was weaker in 85 % of the tamoxifen-selected clones compared to the parent cell line

**Fig. 3:**
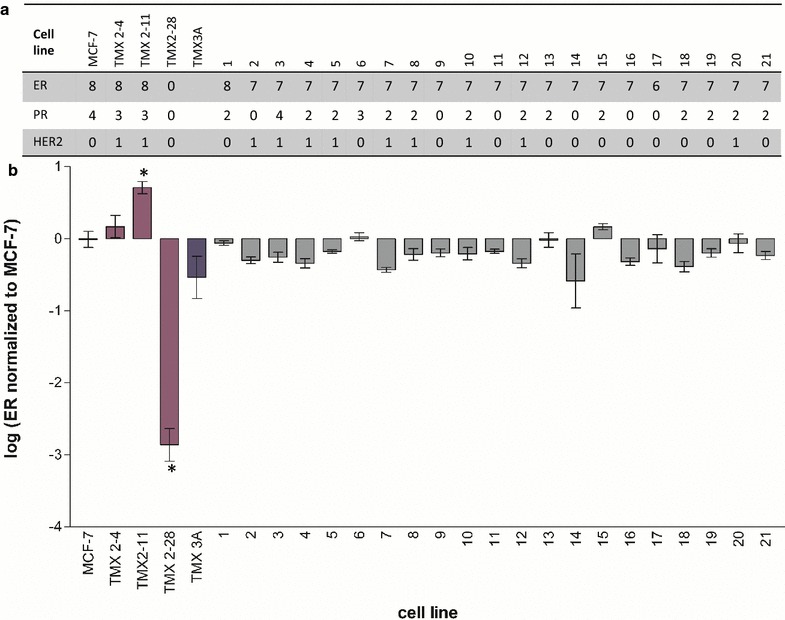
Quantification of hormone receptor status in tamoxifen-selected cell lines. **a** Scores for parent cell line (MCF-7), previously established tamoxifen-resistant cell lines (TMX2-4, TMX2-11 and TMX2-28) and the new tamoxifen-selected clones (1–21). **b** Relative expression of ER for parent cell line (MCF-7), previously established tamoxifen-resistant cell lines (TMX2-4, TMX2-11 and TMX2-28) and the new tamoxifen-selected clones (1–21). Analysis was conducted on ER expression levels normalized to the reference gene HPRT. Data are shown as log of the ratio of the tamoxifen-selected cell line to MCF-7. *Error bars* indicate the standard deviation; N = 2 biological replicates run in technical duplicate; *p < 0.001; Results for TMX2-28 have been published previously [13, 23]

Blinded observers (pathologists RMJ and CNO) assessed IHC-stained slides for ER, PR and HER2. ER protein expression in the 21 tamoxifen-selected cell lines was similar to that observed in the parental MCF-7 (Figs. 2, 3a). Nineteen of the tamoxifen-selected cell lines received an Allred score of 7, and one line each received an Allred score of 5 and 6, which is only slightly higher than the score of 6 routinely assigned ER staining in MCF-7 [22]. In contrast, the protein expression for PR was substantially lower in the tamoxifen-selected cell lines than in the parental MCF-7 (Figs. 2, 3a). For progesterone receptor, six cell lines received an Allred score of 0, thirteen lines received a score of 2, and one line each received a score of 3 and 4. Allred scores of two and less are considered PR-negative. Thus, only two of the 21 cell lines were scored positive for PR and these positive scores are still considerably lower than the typical Allred score of 6 assigned for PR staining in MCF-7 [22]. HER2 protein expression was similar between the parental and tamoxifen-selected cell lines with all lines receiving a score of 0 or 1+ (Figs. 2, 3a).

### Comparison of mRNA levels in parental, novel and previously established tamoxifen-selected cell lines

Having established with IHC that the novel tamoxifen-selected cell lines were ER-positive, PR-negative (18 of 21), and HER2-negative, we wanted to examine the expression of additional genes that might play a role in endocrine resistance, and compare the expression in the novel cell lines with the expression in several previously described tamoxifen-resistant cell lines, TMX2-28, TMX2-11, and TMX2-4 [13, 23]. We began with a comparison of ESR1 mRNA levels to confirm the ER-immunostaining data. As expected, based on the protein data, the ESR1 mRNA levels of the novel tamoxifen-selected cell lines did not differ significantly from the parental MCF-7 (Fig. 3b). Interestingly, however, the ESR1 mRNA levels for the majority of the cell lines were slightly lower than that of MCF-7. In comparison, ESR1 expression is significantly higher in TMX2-11 as compared to MCF-7 (p < 0.001) and significantly lower in TMX2-28 as compared to MCF-7 (p < 0.001), confirming previous reports [13, 23].

**Fig. 4:**
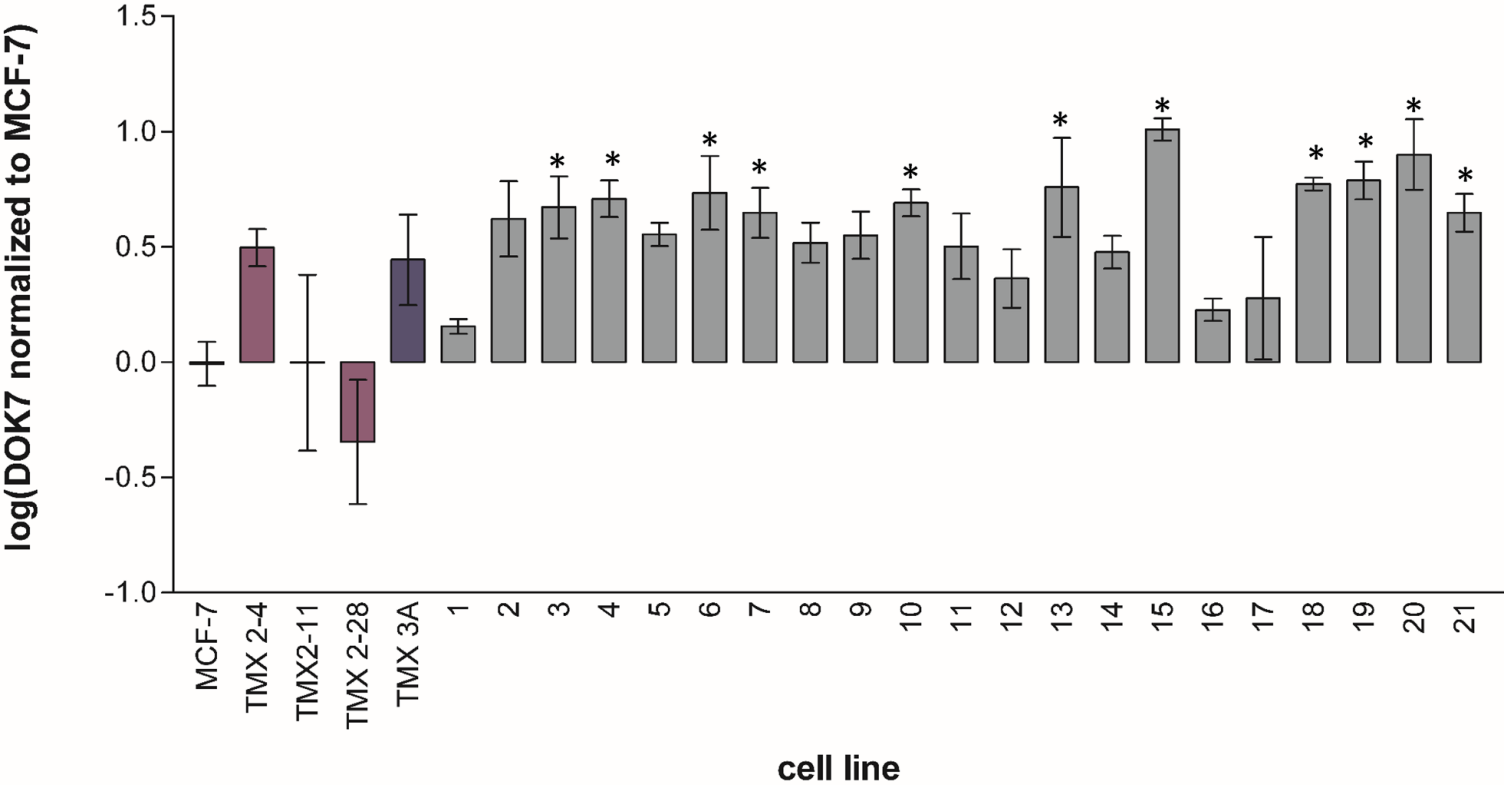
Relative expression of DOK7 in the 21 clonal cell lines compared to that of MCF-7 and the established TMX 2-28, TMX 2-11, and TMX 2-4. Analysis was conducted on DOK7 expression levels normalized to the reference gene HPRT. Data are shown as log of the ratio of the tamoxifen-selected cell line to MCF-7. *Error bars* indicate the standard deviation; N = 2 biological replicates run in technical duplicate; *p < 0.05

**Fig. 5:**
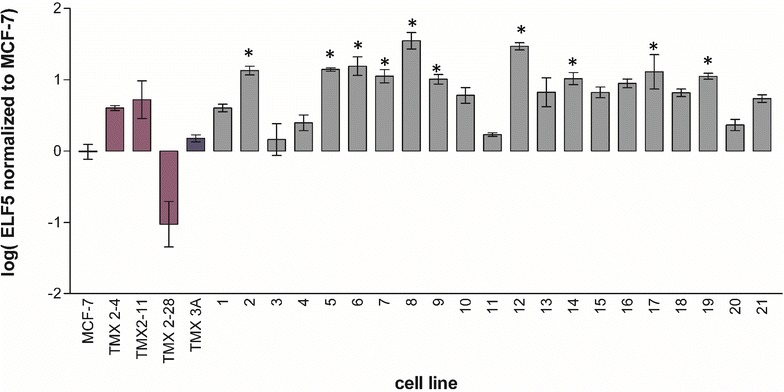
Relative expression of ELF5 in the 21 clonal cell lines compared to that of MCF-7 and the established TMX 2-28, TMX 2-11, and TMX 2-4. Analysis was conducted on ELF5 expression levels normalized to the reference gene HPRT. Data are shown as log of the ratio of the tamoxifen-selected cell line to MCF-7. *Error bars* indicate the standard deviation; N = 2 biological replicates run in technical duplicate; *p < 0.05

We next compared the expression of DOK7 mRNA in MCF-7 with the expression in all of the tamoxifen-selected lines and the mass culture TMX3A. DOK7 was significantly overexpressed in eleven of the 21 novel tamoxifen-selected cell lines (p < 0.05) and was not significantly altered in any of the other cell lines or in the mass culture (Fig. 4). Interestingly, DOK7 expression was reduced, although not significantly, in TMX2-28 the only ER-negative tamoxifen-resistant cell line examined. Next, we compared ELF5 expression in MCF-7 with the expression in all of the tamoxifen-selected lines and the mass culture. ELF5 was significantly over expressed in ten of the 21 novel cell lines (p < 0.01) and not in the mass culture or the previously described cell lines (Fig. 5). Again, expression was decreased, but not significantly, in the ER-negative TMX2-28 cell line. There was limited overlap between the tamoxifen-selected cell lines with elevated DOK7 mRNA levels and those with elevated ELF5 mRNA levels; only three cell lines (clones 6, 7 and 19) had increased expression of both genes.

### DNA Promoter methylation of DOK7 and ELF5 in cell lines

Given that the expression of DOK7 and ELF5 was significantly increased in roughly half of the novel tamoxifen-selected, ER–positive cell lines, and decreased (although not significantly) in the only ER-negative cell line, TMX2-28, we wanted to determine the extent to which DNA methylation in the promoter region of DOK7 and ELF5 controlled gene expression. If DNA promoter methylation was controlling expression we would expect to see a *decrease* in methylation associated with the up-regulation observed in the novel tamoxifen-selected cell lines, and an *increase* in DNA methylation associated with the down-regulation observed in the TMX2-28 cell line.

**Fig. 6:**
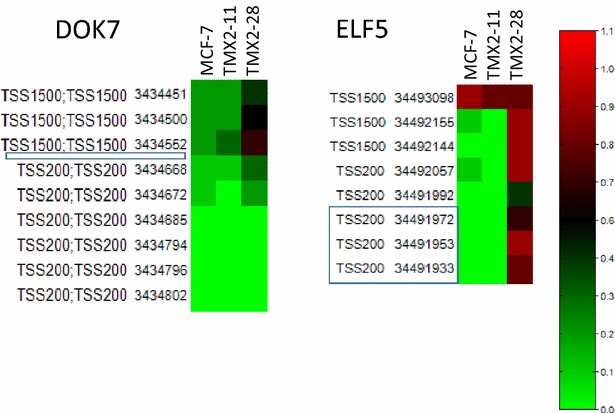
Promoter methylation in MCF-7 cells compared with the tamoxifen-resistant cell lines, TMX2-11 and TMX2-28. Heat maps were prepared from available HM450BC data [12]. Average beta values, coordinate 36 values, and location are shown for the CpGs within the TSS200 and TSS1500 regions. *Left panel*: nine CpGs within the TSS of DOK7 are included on the HM450BC; the *box* indicates the location of the six CpGs examined with pyrosequencing, none of which were present on the bead chip (see Fig. 7). *Right panel*: eight CpGs within the TSS of ELF5 are included on the HM450BC; the *box* indicates the location of the four CpGs that were examined with pyrosequencing, three of which were also present on the bead chip (see Fig. 7)

We first examined DNA methylation data available from the Illumina human methylation 450K bead chip (HM450BC) for MCF-7, TMX2-11 and TMX2-28 [12]. The HM450BC includes nine CpGs within the transcriptional start site (TSS) of DOK7, and eight CpGs within the TSS-region of ELF5. As shown in the heat maps of Fig. 6, the methylation in the TSS region of both DOK7 and ELF5 are substantially increased in TMX2-28 cells as compared to MCF-7 and TMX2-11. The increase in methylation observed in TMX2-28 cells is consistent with the lower expression of DOK7 and ELF5 in this cell line and suggests that promoter methylation in DOK7 and ELF5 may control gene expression. The results from TMX2-28 are in agreement with the inverse association between promoter methylation and gene expression previously demonstrated for ELF5 [24–26] and DOK7 [27]. In contrast, the increased expression of both ELF5 and DOK7 observed among a subset of the ER-positive, tamoxifen-selected, cell lines cannot be attributed to a decrease in promoter methylation as compared to the parental cell line, since the parental MCF-7 methylation is already near zero at the majority of tested sites.

**Fig. 7:**
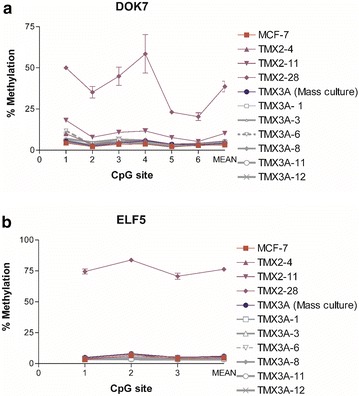
Confirmation of promoter methylation observed in the HM450BC and analysis of methylation in the novel TMX3A tamoxifen-selected mass culture, and a sample of tamoxifen-selected clonal cell lines. Pyrosequencing results of bisulfite-modified DNA are shown for the genes **a** DOK7 and **b** ELF5. Mean and standard deviations for technical replicates (N = 2) are shown for each of the CpGs interrogated and for the mean of the CpGs

To confirm the methylation results observed with the HM450BC we designed pyrosequencing assays to interrogate six CpG sites within the TSS of DOK7 and three CpG sites within the TSS of ELF5. Figure 7a shows the pyrosequencing results for MCF-7, TMX2-4, TMX2-11, TMX2-28, the mass culture TMX3A and several TMX3A clonal cell lines for DOK7. As expected, only the ER-negative TMX2-28 cell line has increased DNA methylation. Average DOK7 methylation in TMX2-28 cells was 35.7 %, whereas average methylation of MCF-7 was 3.3 % and TMX3A was 4.2 %. Pyrosequencing of all 21 novel tamoxifen-selected cell lines showed no differences in DNA methylation in DOK7 as compared to the parent MCF-7 (6 representative tamoxifen-selected cell lines are shown).

Likewise, increased DNA methylation of ELF5 was observed only for TMX2-28 (Fig. 7b). Average DNA methylation of ELF5 in TMX2-28 was 74.9 % compared to MCF-7 at 5.5 %, and TMX2-4, 2-11 and TMX3A (mass culture) at 4.5, 5.75, and 6 %, respectively. Additionally, all 21 tamoxifen-selected clonal cell lines, six of which are shown in Fig. 7b, had low methylation of ELF5.

### DNA methylation of DOK7 and ELF5 in paired primary and second breast tumors

We next examined DNA methylation in primary and second/recurrent breast tumors from three groups of women (Table 1) to determine the extent to which the tamoxifen-related changes observed in cell culture also occurred in the tumors of women treated with anti-hormonal therapy. Primary and recurrent tumors were categorized by PR status because PR expression is an indication of ER activity [28]. Furthermore, in the majority of novel tamoxifen-selected clones described in this report, decreases in PR expression were accompanied by increases in ELF5 and DOK7 expression. As shown in Table 1, all 16 women who had a PR-positive primary tumor received hormonal therapy, and 10 of the 16 are known to have received tamoxifen (primary tumors from groups 1 and 2: tamoxifen 10; aromatase inhibitors 2; type of hormonal therapy unknown 4). Additionally, 3 of the 8 women with PR-negative primary tumors (group 3) received hormonal therapy because their primary tumor was ER-positive.

**Table 1:** Demographics and tumor information for primary and second tumors stratified by progesterone receptor (PR) status

Group	Primary tumor	Second tumor
(1) PR+ to PR+ (n = 10)		
Age (±SD), range	53.8 (±16.9), 37–84	61.4 (±16.7), 40–90
Tumor type	DCIS 1; IDC 8; ILC 1	DCIS 1; IDC 7; ILC 2
Hormonal therapy	Yes = 10 (6 tamoxifen, 4 type unknown)	
(2) PR+ to PR− (n = 6)		
Age (±SD), range	54 (±7.8), 42–62	63.3 (±7.1), 55–74
Tumor type	DCIS 1; IDC 3; ILC 1; IDC and ILC 1	DCIS 2; IDC 3; ILC 1
Hormonal therapy	Yes = 6 (4 tamoxifen, 2 aromatase inhibitor)	
(3) PR− to PR− (n = 8)		
Age (±SD), range	60.1 (±10.6), 46–79	63.3 (±11.1), 48–80
Tumor type	DCIS 1; IDC 7	DCIS 1; IDC 7
Hormonal therapy	No = 5; Yes = 3 (all tamoxifen)	

*DCIS* ductal carcinoma in situ, *IDC* invasive ductal carcinoma, *ILC* invasive lobular carcinoma

**Fig. 8:**
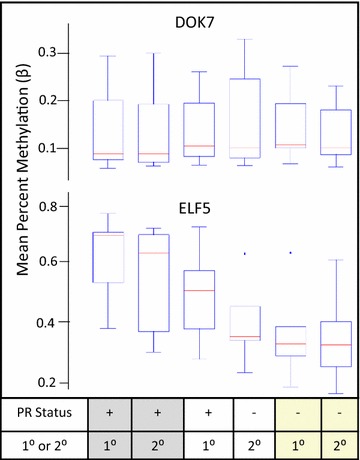
Promoter methylation of DOK 7 and ELF5 in primary (1°) and second or recurrent (2°) breast tumors by PR status of three groups of women: women who had a primary PR-positive tumor and a second PR-positive tumor (n = 10, *gray boxes*); women who had a primary PR-positive tumor and a second PR-negative tumor (n = 6; *white boxes*); and women who had a primary PR-negative tumor and a second PR-negative tumor (n = 8; *yellow boxes*) Average beta distribution analysis of 9 CpG sites in the TSS of DOK7 (*top*) and 8 CpGs sites in the TSS of ELF5 (*bottom*). *Box* is quartile 1 and 3; *red line* is median average β value, *whiskers* are the highest and lowest values within ×1.5 interquartile range

**Fig. 9:**
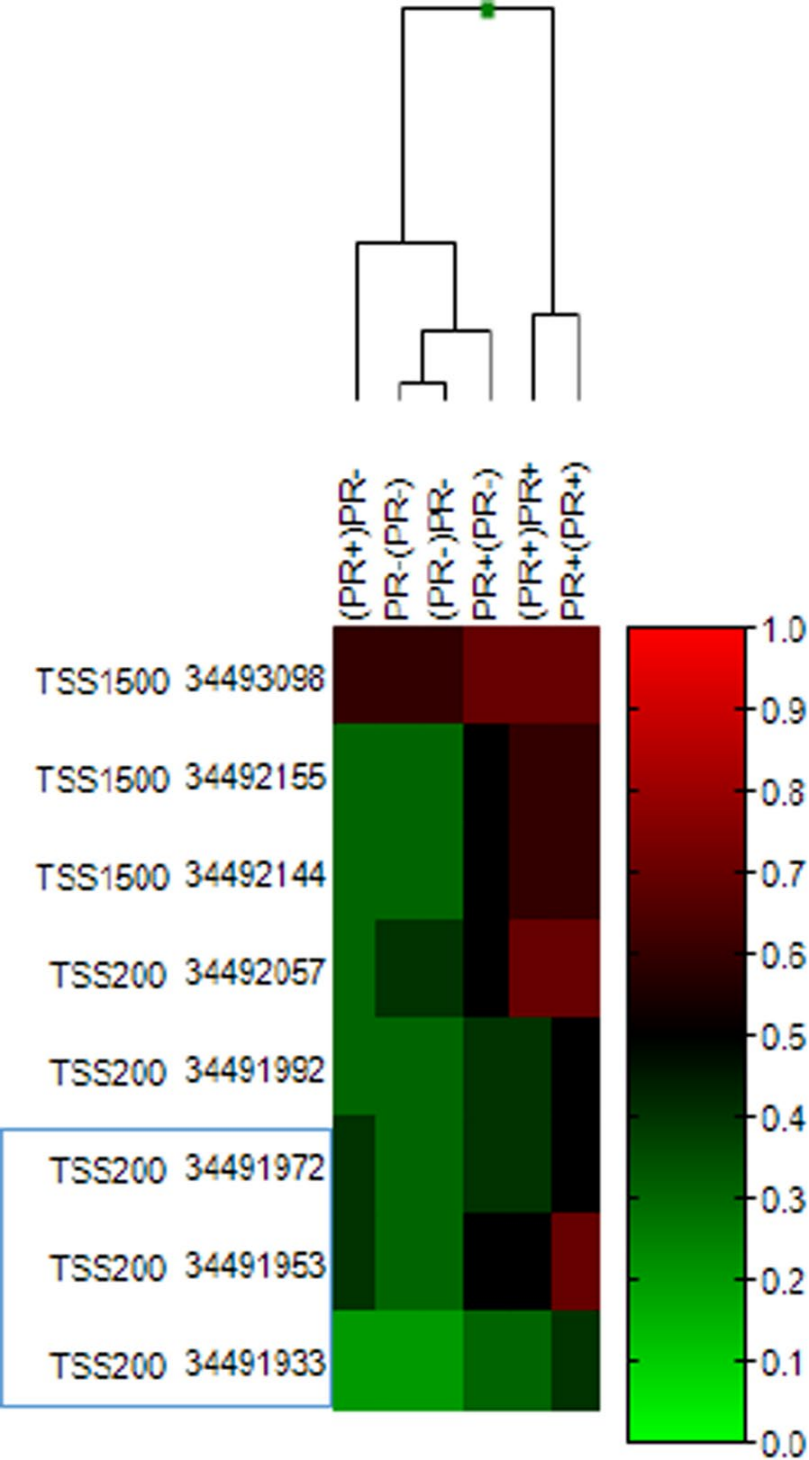
Cluster analysis of CpGs within the TSS1500–TSS200 in ELF5 for primary and recurrent tumors from the three groups of women described in Fig. 8. The PR status of the primary tumor is followed by the PR status of the recurrent tumor, and the data shown are for the tumor group not within the primary parentheses; e.g., (PR+)PR− indicates that the data shown are for the PR-negative second tumors from the group of women who had a PR-positive primary and PR-negative recurrence. *Blue box* indicates CpG sites that were interrogated by pyrosequencing (see Fig. 7). Coordinate 36 values and functional genomic location are shown

Comparison of DOK7 promoter methylation across the primary and second tumors of women in the three groups showed no differences (Fig. 8a); median mean methylation for the 9 CpGs sites within the TSS was below 10 % in all groups. In contrast, analysis of the 8 CpGs in the TSS of ELF5 showed wide differences among groups (Fig. 8b). First, ELF5 was significantly more methylated in PR-positive tumors than in PR-negative tumors (β = .58 and .39, respectively; t = 4.97; df = 45; p < 0.001). The lower methylation of ELF5 in PR-negative tumors is consistent with the increased expression of ELF5 observed in ten of our tamoxifen-selected cell lines, all of which had decreased levels of PR. Second, there was a trend for primary tumors from women treated with anti-estrogen therapy to have higher mean ELF5 methylation as compared to the woman’s second/recurrent tumor (β = .55 and .49, respectively; paired t = 1.53; df = 19; p = 0.144). This is the first demonstration of a decrease in ELF5 methylation in human breast tumors occurring after anti-estrogen treatment, and is consistent with a report by Kalyuga et al. showing an increase in ELF5 expression in luminal cell lines that acquired resistance to tamoxifen [8]. Of particular interest is the difference between PR-positive primary tumors in women of groups 1 and 2 (Table 1). PR-positive primary tumors from women who had second PR-positive tumors had significantly higher mean methylation than did PR-positive primary tumors from women whose second tumor was PR-negative (β = .64 and .51, respectively; t = 3.54; df = 14; p = 0.003). To further examine the relationship between ELF5 methylation, PR status, and tumor occurrence, cluster analysis was performed with GenomeStudio using a correlation matrix (Fig. 9). Groups from women whose first and recurrent tumors were both PR-positive clustered together (far right). Groups from first and recurrent tumors of women where the recurrent tumor was PR-negative clustered separately. The ELF5 methylation of PR-positive primary tumors in women who went on to have a PR-negative recurrence was clearly distinct from the ELF5 methylation of other PR-positive tumors. This indicates the possibility of stratification of women for tailored treatment in the early stages of disease.

Widespread resistance to endocrine therapies limits their usefulness and reduces patient remission. Previous studies have identified tamoxifen-induced cellular changes. Here we characterize novel clonal cell lines whose diversity could represent drug resistant cancers in women. All of the tamoxifen-selected lines remained ER positive, which is consistent with results from past experiments [7, 13], yet most of the lines lost expression of PR. We show that many of these cell lines had significantly elevated DOK7 and ELF5 gene expression compared to MCF-7 cells not treated with tamoxifen. Promoter methylation of DOK7 and ELF5 in the MCF-7 control cell culture was extremely low, and therefore, a further decrease in promoter methylation of DOK7 and ELF5 was not observed to associate with the increased expression seen in the tamoxifen-selected cell lines.

However, we found the promoter region of ELF5 to be highly methylated in primary PR-positive tumors and that methylation was vastly reduced in second tumors occurring after anti-estrogen treatment. This finding is consistent with Kalyuga et al. [8] who found higher expression of ELF5 to correlate with resistance to anti-estrogens. Importantly, we also observed significantly higher levels of ELF5 methylation in the subset of primary, PR-positive tumors that would go on to recur as PR-negative, indicating an opportunity for the development of more personalized treatment in the early stages of disease. To our knowledge this is the first report of differential ELF5 methylation in paired primary and recurrent breast tumors and these results deserve to be replicated in a larger study.

## Conclusions

The novel tamoxifen-resistant cell lines described here display molecular differences from the parental MCF-7 that include decreased PR protein levels, increased ELF5 and DOK7 gene expression and will likely be useful in further mechanistic studies of endocrine-resistance. Examination of paired primary and second tumors from 24 women, 19 of whom received anti-estrogen treatment, revealed a subset of primary PR-positive tumors with distinct ELF5 promoter methylation indicating the possibility of stratification of women for tailored treatment in the early stages of disease.

## Methods

### Generation of tamoxifen-selected cell lines

MCF-7 cells were purchased from the American Type Culture Collection (ATCC) and grown as previously described [23], with tamoxifen added at 1 μM. Briefly, cells were maintained at 37 °C with 5 % CO_2_. Cells were grown in Falcon T-75 flasks, given fresh media twice per week, and split once or twice per week when 70–90 % confluency was obtained. The tamoxifen-selection was conducted in triplicate for six months resulting in three populations (referred to as mass cultures), one of which, TMX3A, was used for generating clonal lines. Cloning by limiting dilution was performed to assess the diversity of cells within the tamoxifen-selected mass culture. Tamoxifen-selected TMX3A cells were seeded at one cell/well and allowed to grow in 96-well plates for 6–7 weeks. Twenty-one cell lines were selected (referred to as clonal cell lines), expanded and cryopreserved.

### Nucleic acid extraction and gene expression

RNA isolation was performed using Tri-reagent (Molecular Research Center, Inc. Cat. No. E8875). DNA was isolated from frozen cells using the QIAmp DNA Mini Kit (Qiagen Cat. No. 51304). RNA and DNA were quantified using a NanoDrop 8000 (Thermo Scientific). cDNA was created from isolated RNA using the High-Capacity cDNA Reverse Transcription Kit (Applied Biosystems) with added RNasin^®^ Ribonuclease Inhibitor (Promega, Ref. N2511). Quantitative RT-PCR (qRT-PCR) was performed on this cDNA using FastStart Universal SYBR Green Master mix (Roche, Cat. No. 04 913 850 001). Primers used for qRT-PCR are listed in Table 2. Primers are designed to span an exon–exon junction, therefore DNaseI treatment of the RNA was not necessary.

**Table 2:** Primers used for q RT PCR

Target	Product size (BP)	Primer sequence (5^′^–3^′^)
Estrogen receptor alpha [13]	204	F: 5′-ATG ATC AAC TGG GCG AAG AG-3′ R: 5′-GAT CTC CAC CAT GCC CTC TA-3′
ELF5 (NM_001243080.1)	129	F: 5′-TGC CCT CAC GGT AAT GTT GGA-3′ R: 5′-TGA TGC TCA AAG GCA GGG TAG-3′
DOK7 (NM_173660.4)	110	F: 5′- GCA GTG GAG GGG ATG ACC-3′ R: 5′ TGA CGA CGA GGA TTG CTC TG-3′

### DNA methylation analyses

500 ng of DNA was bisulfite treated using the EZ DNA methylation-Lightning Kit (Zymo Research Cat # D5030). Bisulfite-treated DNA was pyrosequenced using the Pyromark Q24 system (Qiagen) and manufacturers protocol (Qiagen), and gene methylation levels were detected as cysteine to thymidine ratios at specific loci. Primers used for pyrosequencing are listed in Table 3. For DOK7, the region interrogated lies within the transcriptional start site region, from chromosome 4: 3434598 to 3434634 (NCBI build36 coordinates). For ELF5, the region interrogated lies within the transcriptional start site region from chromosome 11 (−1): 34491972 to 37791933 (NCBI build 36 coordinates).

**Table 3:** Primers used for pyrosequencing

Target	Product size (BP)	Pyrosequencing primer sequences (5′–3′)
ELF5	230	F: 5′-GTT TGT AGG GTA GGG GTG AGT T-3′ R: 5′-BIOTIN-ACA AAC CCT CCC AAC ACC A-3′ SEQ: 5′-TAA GGA GTAGTG TTA TAT TG-3′
DOK7	265	F: 5′- GGG AAG TAG GAT TGT TTG AAG ATT -3′ R: 5′- BIOTIN-CCA ACC AAA CTC CTT CTC CTA-3′ SEQ: 5′-GTT TTT GTT TTT TGA AAA AG-3′

### Immunohistochemistry and scoring for ER, PR and HER2

Cells were seeded onto poly-l-lysine coated-slides (Polysciences #22247) at a density of 200,000 cells/mL/slide and fixed the following day using 100 % ice-cold methanol. Slides were stained on a BenchMark Ultra platform using the UltraView Universal DAB Detection Kit with previously optimized antibodies for ER (Ventana CONFIRM anti estrogen receptor SP1 Rabbit monoclonal primary antibody), PR (Ventana CONFIRM anti progesterone receptor 1E2 Rabbit monoclonal primary antibody) and HER2 (Ventana PATHWAY anti HER-2/neu antibody 4B5 Rabbit monoclonal antibody). Stained slides were dehydrated in ethanol and xylene and coverslips were added manually. Slides were examined by a pathologist and ER and PR were recorded as Allred scores [29], HER2 was scored positive if greater than 30 % of the cells showed 3+ membrane staining; scores of 0–2 were considered negative [22].

### Human breast tumors

Collection and processing of human breast tumors was conducted with IRB approval from Baystate Medical Center and the University of Massachusetts as previously described [30]. Breast tumor samples purified using the BiOstic FFPE tissue DNA isolation kit (Mo Bio, Carlsbad, CA) were sent to the core facility at the University of Southern California for HM450 BeadChip (Illumina) analysis and detailed results are in preparation (Williams in prep). Twenty-four matched pairs of primary and second/recurrent breast tumors for which methylation data and hormone receptor status were known and for which patient treatment data, and age were available were selected. We use the term second and recurrent interchangeably throughout the manuscript. It is unknown whether the second tumor is a true new second tumor or a recurrence of the first tumor. Nineteen of the 24 women received anti-estrogen therapy after resection of the first tumor, i.e. prior to resection of the second tumor (Table 1).

### Data analysis

Q RT-PCR data represent biological duplicates for each cell line run in technical duplicate. The data were normalized to hypoxanthine-guanine phosphoribosyltransferase (HPRT) as a reference gene. One-way ANOVAs with Bonferroni post hoc tests were run to determine significance at p < 0.05. For presentation purposes, each value was normalized to the average MCF-7 value for the gene of interest. Data were then log transformed to provide the expression levels relative to MCF-7, with values greater than 0 indicating higher expression, and values below 0 indicating reduced expression. The average of 4 biological replicates was used to determine the standard deviation. Error bars on Figs. 3, 4, 5 and 7 represent the standard deviation. Data were analyzed using GraphPad Prism (version 3.02, La Jolla, CA). GenomeStudio Methylation Module (v.1.9, Illumina, San Diego, CA) was used to analyze the β values of the methylation data obtained from the HM450 BeadChip. Statistical comparisons between groups were conducted with paired and unpaired t-tests provided in the Data Analysis ToolPak of Excel (Microsoft Office Professional plus 2013).

## Abbreviations

CpG: cytosine-phosphodiester bonded to a Guanine
ELF5: E74-like factor 5
ER: estrogen receptor
HER2: human epidermal growth factor receptor 2
HM450BC: human methylation 450K bead chip
HPRT: hypoxanthine–guanine phosphoribosyltransferase
IHC: immunohistochemistry
PR: progesterone receptor
qRT-PCR: quantitative reverse transcriptase polymerase chain reaction
TSS1500: transcriptional start site 1500 region
TSS200: transcriptional start site 200 region
